# NF-κB-modulated miR-130a targets TNF-α in cervical cancer cells

**DOI:** 10.1186/1479-5876-12-155

**Published:** 2014-06-01

**Authors:** Jian Zhang, Haidong Wu, Pu Li, Yanzheng Zhao, Min Liu, Hua Tang

**Affiliations:** 1 Tianjin Life Science Research Center and School of Basic Medical Sciences, Tianjin Medical University, No. 22 Qi-Xiang-Tai Road, Tianjin 300070, China; 2 Tianjin Central Hospital of Gynecology Obstetrics, Tianjin, China

**Keywords:** miRNA, miRNA-130a, NF-κB, Cervical cancer, TNF-α

## Abstract

**Background:**

Nuclear factor-κB (NF-κB) induces a variety of biological processes through transcriptional gene control whose products are components in various signaling pathways. MicroRNAs are a small endogenous non-coding RNAs that regulate gene expression and are involved in tumorigenesis. Using human cervical cancer cell lines, this study aimed to investigate whether NF-κB could regulate miR-130a expression and the functions and targets of miR-130a.

**Methods:**

We used the HeLa and C_33_A cervical cancer cell lines that were transfected with NF-κB or miR-130a overexpression plasmids to evaluate their effects on cell growth. We utilized bioinformatics, a fluorescent reporter assay, qRT-PCR and Western blotting to identify downstream target genes.

**Results:**

In HeLa and C_33_A cells, NF-κB and miR-130a overexpression promoted cell growth, but genetic knockdowns suppressed growth. TNF-α was identified as a target of miR-130a by binding in a 3’-untranslated region (3’UTR) EGFP reporter assay and by Western blot analysis. Furthermore, low TNF-α concentrations stimulated NF-κB activity and then induced miR-130a expression, and TNF-α overexpression rescued the effects of miR-130a on cervical cancer cells.

**Conclusions:**

Our findings indicate that TNF-α can activate NF-κB activity, which can reduce miR-130a expression, and that miR-130a targets and downregulates TNF-α expression. Hence, we shed light on the negative feedback regulation of NF-κB/miR-130a/TNF-α/NF-κB in cervical cancer and may provide insight into the carcinogenesis of cervical cancer.

## Background

Nuclear factor-κB (NF-κB) is a nuclear transcription factor that regulates the expression of a large number of genes associated with inflammation [1,2], tissue damage and repair [3,4], cell differentiation [5,6], apoptosis [7,8] and tumor growth [9,10]. NF-κB is composed of five distinct but structurally related subunits: RelA, RelB, c-Rel, p50 and p52, and these five mature proteins can form various homodimeric and heterodimeric combinations [11,12]. The dimers remain in an inactive form in the cytoplasm, sequestered by one of the IκB family members, mainly IκBα. In canonical NF-κB signaling, nuclear translocation of NF-κB is controlled by the signal-induced degradation of IκBs. Exposure of cells to stimuli, such as pro-inflammatory cytokines, activates the IκB kinase (IKK), which phosphorylates IκB, and IκB then becomes susceptible to ubiquitination and degradation through the proteasome system. Free p50 and p65 or c-Rel then translocate into the nucleus to activate related genes, and this gene regulation occurs mainly through promoter element binding [13,14]. Some pro-inflammatory cytokines, chemokines, and many oncogenes associated with tumor development and progression are NF-κB activators, including palmitic acid, the most abundant long chain saturated fatty acid [15]. Furthermore, the mitochondrial antiviral signaling (MAVS) protein can recruit several TRAF proteins, and these proteins are associated with NF-κB activation [16]. Another protein, protein-arginine methyltransferase 5 (PRMT5), is overexpressed in many cancers and promotes tumorigenesis by stimulating NF-κB [17]. Though these activators, TNF-α acts as an important proinflammatory cytokine to stimulate NF-κB activity.

TNF-α, one form of TNF [18], acts as a proinflammatory cytokine, and increased TNF-α levels have been observed in serum and other patient samples with inflammation [19]. It is mainly produced by monocytes, macrophages, initial hemorrhages and necrosis of tumor tissue [20,21]. TNF-α is a secreted protein, mature TNF-α protein plays its role mainly through its receptors on the surface [22-24]. So far, TNF-α seem powerful to destroy tumor, but has fallen short of expectations in clinical use as an anti-tumor agent because of its indeterminacy at therapeutic doses. The autocrine or paracrine TNF-α expression can make the opposite effect, like some reports showed TNF-α play a role in the promotion of cell growth in low concentration [25,26]. Low doses of endogenous TNF-α produced by normal epithelial cells or epithelial-derived cancer cells can also act as a tumor promoter, in NSCLC cells, the TNF-α expression may affect the normal lung adjacent to the tumor [27], in human breast cancer cell, TNF-α enhanced invasiveness of the malignant cells dependent on matrix metalloprotease [28], in ovarian cancer cells the CXCR4 expression also in a TNF-α–dependent manner [29]. Many studies have demonstrated the role of TNF-α in cell proliferation, and some reports have shown that TNF-α acts as an apoptotic mediator in some cell lines, such as hematopoietic cells [30] and cartilage progenitor cells [31]. This proapoptotic effect can be weakened by other substances in the body, such as miRNAs. However, little is known about the ability of miRNAs to regulate TNF-α expression.

miRNAs are small non-coding RNAs that regulate gene expression at the post-transcriptional level through translational repression or mRNA degradation. They exert control over approximately 60% of human genes associated with development, cell differentiation, growth, motility and apoptosis [32,33], which render miRNAs one of the most abundant classes of regulatory molecules. Particularly in malignancies, aberrant miRNA expression has emerged as a hallmark of cancer [34]. Because each miRNA is thought to regulate hundreds of mRNAs, and each mRNA is also modulated by many miRNAs [35], the identification of miRNA targets represents an important step in understanding miRNA function. miR-130a, a miRNA has been shown to promote cell survival in several cell lines through different signaling mechanisms [36-38], but its mechanism of action and expression are still not clear in cervical cancer.

In this study, we found that NF-κB and miR-130a promoted cervical cancer cell growth. miR-130a directly targeted the 3’UTR of TNF-α and repressed its translation, and TNF-α activated NF-κB to upregulate miR-130a expression. Thus, TNF-α can stimulate NF-κB activity and enhance miR-130a levels, which then reduces TNF-α levels. Therefore, this NF-κB/miR-130a/TNF-a/NF-κB feedback signaling pathway may play an important role in the growth regulation of cervical cancer cells.

## Materials and methods

### Cell culture and transfection

The human cervical cancer cell lines HeLa and C_33_A were grown in RPMI1640 medium (GIBCO BRL, Grand Island, NY, USA) supplemented with 10% fetal bovine serum (FBS), 100 IU/ml of penicillin and 100 mg/ml of streptomycin. The cell lines were incubated at 37°C in a humidified chamber supplemented with 5% CO_2_. Transfections were performed using the Lipofectamine 2000 Reagent (Invitrogen, Carlsbad, CA, USA) according to the manufacturer’s instructions. All transfections were performed in three independent experiments.

### Bioinformatics

miRNA targets were predicted using the following algorithms: Target-Scan, PicTar, and MiRBase Targets.

### Plasmid construction

To construct the pcDNA3/NF-κB vector expressing p50 subunit, we amplified a DNA fragment carrying p50 by PCR using sense and antisense NF-κB primers, and then the fragment was cloned into the pcDNA3 vector at the EcoRI and Xhol restriction sites.

To construct the pSilencer/shR-NF-κB vector interfere p50 subunit expression, a 70-bp double-stranded fragment was obtained via an annealing reaction using two single-strands. The fragment was then cloned into a pSilencer 2.1 vector plasmid (Ambion) at the BamHI and Hind III sites.

To construct a pcDNA3/pri-miR-130a vector expressing miR-130a, we amplified a DNA fragment carrying pri-miR-130a from genomic DNA using sense and antisense Pri-130a primers. The 3’UTR, including predicted target sites, was amplified by PCR using TNF-α-3’UTR-sense and -antisense primers, and the amplified sequence was cloned into an expression plasmid (pcDNA3/EGFP) downstream of the EGFP gene between the EcoRI and BamHI restriction sites. Similarly, the 3’UTR containing mutated miR-130a binding sites was amplified using PCR site-directed mutagenesis and cloned into the pcDNA3/EGFP plasmid between the same restriction sites with TNF-α-3’UTR-mut-sense and -antisense primers. The resulting vectors were designated as pcDNA3/EGFP-TNF-α-3’UTR and pcDNA3/EGFP-TNF-α-3’UTRmut.

The human TNF-α mRNA lacking the 3’UTR was amplified from human HeLa cell cDNA by PCR using sense and antisense TNF-α primers, which are listed in Table 1, and then cloned into the pcDNA3 vector at the EcoRI and Xhol restriction sites, which created the pcDNA3/TNF-α plasmid.

**Table 1:** Oligonucleotide sequences

**Name**	**Sequence (5’-3’)**
NF-κB-sence	CGGAATTCGCCACCAGAATGGCAGAAGATGATC
NF-κB-antisence	TGTCACTCGAGGCAATTTTGCCTTCTAGAGGTC
NF-κB-siR-Top	GATCCCGCCTGAACAAATGTTTCATTTGGTCAAGAGCCAAATGAAACATTTGTTCAGGCTTTTTTGGAAA
NF-κB-siR-Bot	AGCTTTTCCAAAAAAGCCTGAACAAATGTTTCATTTGGCTCTTGACCAAATGAAACATTTGTTCAGGCGG
Pri-130a-sense	ATGGGATCCAGAGGGAGCCCGTGAGCTG
Pri-130a-antisense	CGGAATTCGTATAACTAACAAGTGAGGCTACC
ASO-miR-130a	AUGCCCUUUUAACAUUGCACUG
ASO-NC	TGACTGTACTGAGACTCGACTG
miR-130a-RT	GTCGTATCCAGTGCAGGGTCCGAGGTGCACTGGATACGACATGCCCT
miR-130a-Forward	TGCGGCAGTGCAATGTTA
U6-RT	GTCGTATCCAGTGCAGGGTCCGAGGTATTCGCACTGGATACGACAAAATATGG
U6- Forward	TGCGGGTGCTCGCTTCGGCAGC
miR-Reverse primer	CCAGTGCAGGGTCCGAGGT
TNF-α-3’UTR-sense	CGGGATCCGAAATTGACACAAGTGGACC
TNF-α-3’UTR-antisense	CGGAATTCCTCCCAAATAAATACATTCATCTG
TNF-α-3’UTR-mut-sense	CCCTCTATTTATGATACGAGTTGTGATTATTT
TNF-α-3’UTR-mut-antisense	AAATAATCACAACTCGTATCATAAATAGAGGG
TNF-α-sense	CGGAATTCGCCACCATGAGCACTGAAAGC
TNF-α-antisense	CCCGCTCGAGGCCAGGGCAATGATCCCAAAG
β-actin-sense	CGTGACATTAAGGAGAAGCTG
β-actin-antisense	CTAGAAGCATTTGCGGTGGAC
GAPDH-sense	GCGAATTCCGTGTCCCCACTGCCAACGTGTC
GAPDH-antisense	GCTACTCGAGTTACTCCTTGGAGGCCATGTGG

All DNA oligonucleotides used are listed in Table 1, and all constructs were confirmed by DNA sequencing.

### Fluorescent reporter assay

HeLa and C_33_A cells were co-transfected with pcDNA3/pri-miR-130a or ASO-miR-130a in a 48-well plate followed by the pcDNA3/EGFP-TNF-α-3’UTR or pcDNA3/EGFP-TNF-α-3’UTR-mut reporter plasmids. The RFP expression vector, pDsRed2-N1 (Clontech, Mountain View, CA), was used for normalization. Cells were lysed with RIPA 48 h after transfection, and proteins were collected. EGFP and RFP fluorescence intensities were determined using an F-4500 fluorescence spectrophotometer (HITACHI, Tokyo, Japan).

### Cell viability assay

HeLa and C_33_A cells were seeded in 24-well plates overnight and then transfected with plasmid (1 μg each) or oligonucleotides (final concentration 200 nM). Afterwards, cells were trypsinized and counted, seeded in 96-well plates (in triplicate) for an MTT assay at a density of 8,000 cells/well (HeLa) or 10,000 cells/well (C_33_A), and incubated at 37°C for 24 h. Then, at 24, 48 and 72 h after cell seeding, 10 μl MTT (final concentration, 0.5 mg/ml) was added, and the cells were maintained at 37°C for another 4 h. The medium was removed, and the precipitate was dissolved in 100 μl DMSO. After shaking for 15 min, the absorbance at 570 nm (A570) was measured using an uQuant Universal Microplate Spectrophotometer (Bio-Tek Instruments, Winooski, USA).

### Colony formation assay

Following transfection in 24-well plates as described above, HeLa and C_33_A cells were counted and seeded in 12-well plates (in triplicate) at a density of 200 cells/well. The plates were incubated at 37°C in a 5% CO_2_ humidified incubator. The culture medium was replaced every 3 days. After 14 days in culture, cells were stained with crystal violet and counted. Colonies with at least 50 cells were considered for quantification.

### RNA preparation and Quantitative reverse transcrption PCR (qRT-PCR)

Total RNA extracted from HeLa and C_33_A cells was using the TRIZOL reagent (Invitrogen, Carlsbad, CA), according to the manufacturer’s instructions. RNA extraction from tissue samples was performed by using the mirVana miRNA Isolation Kit (Ambion) according to the manufacturer’s instructions. To assess RNA integrity, a portion each RNA sample was used for concentration and purity measurements (A260 and A280 by spectrophotometry), and a separate portion was subjected to denaturing electrophoresis in a 1% agarose gel stained with ethidium bromide.

qRT-PCR used to detect the miRNA and mRNA levels and was performed using an iQ5 real-time PCR detection system (Bio-Rad). Moloney murine leukemia virus (M-MLV) reverse transcriptase (Promega, Madison, WI) was used to reverse transcribe cDNA from RNA samples. The SYBR Premix Ex Taq™ kit (TaKaRa, Otsu, Shiga, Japan) was used to measure amplified DNA. Related primers were purchased from AuGCT, Inc. (Beijing, China), and sequences are shown in Table 1.

To detect miR-130a, 2 μg of total RNA (in triplicate) was reverse transcribed to cDNA using M-MLV. U6 was used as a housekeeping gene to normalize gene expression. The following PCR cycles were used: 94°C for 3 min followed by 40 cycles of 94°C for 30 s, 56°C for 30 s, and 72°C for 30 s. To detect target genes, 5 μg of total RNA (in triplicate) was reverse transcribed into cDNA. Endogenous β-actin gene levels were used as a control to normalize gene expression. The following PCR cycles were used: 94°C for 3 min followed by 40 cycles of 94°C for 30 s, 58°C for 30 s, and 72°C for 30 s.

### Western blot

Western blotting was performed to determine protein expression. Briefly, HeLa and C_33_A cells were transfected with plasmid (4 μg each) or control oligonucleotides (final concentration of 200 nM). After seeding in 25-ml culture flasks, cells were washed with PBS and lysed after 30 min in RIPA lysis buffer at 4°C to harvest total protein. To collect nucleoproteins, cells were harvested with PBS, centrifuged to obtain a cell precipitate, 0.4% NP-40 added, and the cell precipitate resuspended. After centrifugation, the supernatant contained the nucleoproteins. Next, 0.1% NP-40 was added to the remaining cell precipitate, the precipitate was resuspended and centrifuged, the supernatant was discarded, and the remaining material resuspended in RIPA buffer to obtain cytoplasmic proteins. Equal amounts of protein were subjected to electrophoresis using a 10% SDS-polyacrylamide gel under denaturing conditions and were then transferred onto a nitrocellulose membrane. To assess protein levels, membranes were incubated in blocking buffer for 4 h at room temperature and then with antibodies raised against p50 or TNF-α overnight at 4°C. Membranes were then washed and subsequently incubated with secondary antibody. Protein expression was assessed by enhanced chemiluminescence and exposure to chemiluminescent film. The LabWorks™ Image Acquisition and Analysis Software (UVP, Upland, CA) were used to quantify the protein band intensities. All the antibodies were purchased from Tianjin Saier Biotech (Tianjin, China).

### Immunofluorescent staining

Cells were seeded on 14-well slides at a concentration of 2000 cells/well and incubated overnight. After the cells adhered to the slides, the culture medium was replaced with medium containing TNF-α (Sigma-Aldrich, St. Louis, MO) at a final concentration of 20 ng/mL, and the cells were cultured for 24 h. Cells were fixed with 4% paraformaldehyde for 30 min, permeabilized with 0.5% Triton X-100 for 5 min, and blocked with 10% donkey serum at room temperature for 2 h. Following blocking, primary antibodies were diluted in 1% normal donkey serum, placed on the slide surface, and incubated at 4°C overnight. Cells were washed, and a FITC-labeled donkey anti-rabbit secondary antibody was diluted in 1% normal donkey serum and added to the slides. Finally, DAPI was used to stain the nuclei. Fluorescent images were acquired using a Digital Sight DS-U1 scanning microscope (Nikon, Tokyo, Japan) at an excitation wavelength of 488 nm. Images were superimposed using the NIS Elements F 2.20 imaging software (Nikon).

### Tumor xenograft experiment

For in vivo tumor study, 3 × 10^6^ Hela cells transfected with pri-miR-130a and it is control vector were suspended in 150 μl of serum-free 1640 for each mouse. Each mouse (6 in each group, female, BALB/c-nu/nu at 6 weeks of age) were implanted subcutaneously in the right armpit of nude mice. All mice were killed 14 days after implantation. The samples were frozen in liquid nitrogen or fixed with phosphate-buffered neutral formalin. All studies were performed under the American Association for the Accreditation of Laboratory Animal Care guidelines for humane treatment of animals and adhered to national and international standards. Ethical approval was given by the medical ethics committee of the Ethics Committees of Tianjin Medical University with the following reference number: TMUhMEC 2011018.

### Immunohistochemistry

The tissue samples were fixed in phosphate-buffered neutral formalin, embedded in paraffin, and cut into 5 μm thick sections. Tissue sections were deparaffinized, rehydrated, and microwave-heated in sodium citrate buffer for antigen retrieval. The sections were then incubated with 0.3% hydrogen peroxide/phosphate-buffered saline for 30 min. Sections were incubated with a primary antibody against TNF-α at a 1:50 dilution and incubated overnight at 4°C. Detection of the primary antibody was performed using goat anti-rabbit-HRP for 1 hour at room temperature and visualized with DAB substrate.

### Statistical analysis

Data are expressed as the mean ± standard deviation (SD). Statistical analyses were performed with a paired t-test. P < 0.05 was considered statistically significant. Experiments were performed in triplicate, and one representative experiment is shown in the Figures.

## Results

### NF-κB promotes the growth of human cervical cancer cells

**Figure 1:**
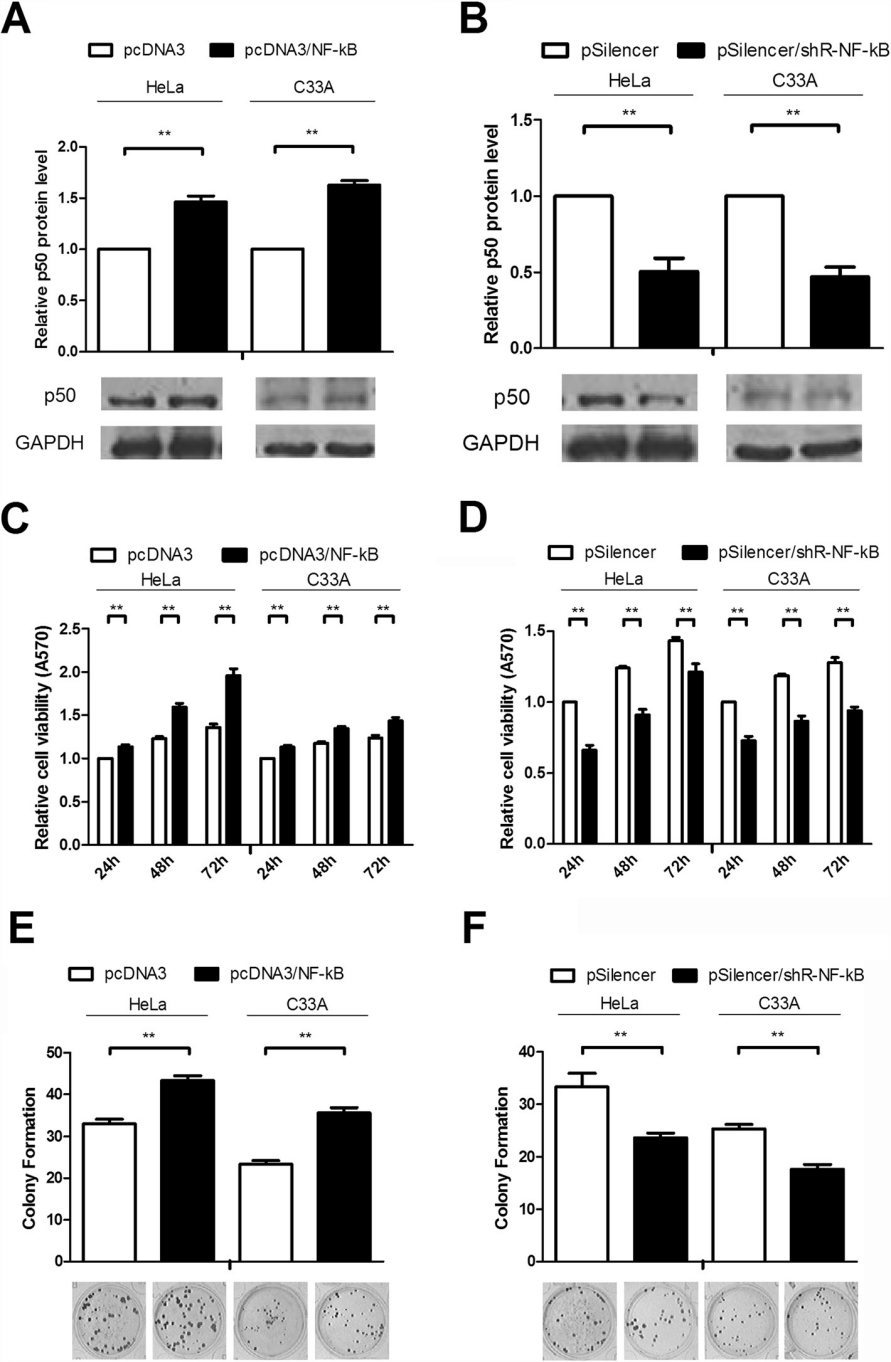
**NF-κB promotes the growth of human cervical cancer cells. (A and B)** Western blot analysis was utilized to detect NF-κB levels in HeLa cells and C_33_A cells transfected with pcDNA3/NF-κB or pSilencer/shR-NF-κB. After transfecting both cell lines, cells were cultured for 48 h, and then lysed for 30 min with RIPA buffer at 4°C. The endogenous GAPDH expression levels were used to normalize protein expression. **(C and D)** The effects of NF-κB on cell viability was assessed by MTT assays. HeLa and C_33_A cells were detached from 24-well plates after transfection with pcDNA3/NF-κB and pSilencer/shR-NF-κB or their respective control vectors. Relative cell growth was assessed at 24, 48 and 72 h after seeding in 96-well plates. **(E and F)** The effects of NF-κB on colony formation. Cells were detached from 24-well plates after transfection and seeded in 12-well plates, and on the 14^th^ day after seeding, the number of colonies was counted. Experiments were performed in triplicate. (*P < 0.05, **P < 0.01).

NF-κB has been shown to promote tumorigenesis, tumor cell proliferation, invasion and metastasis [39]. To investigate the role of NF-κB in cervical cancer cells, we constructed an NF-κB overexpression vector (pcDNA3/NF-κB) and an NF-κB interference vector (pSilencer/shR-NF-κB) which over express or knock down p50 protein that were validated by western blot in transfected HeLa and C_33_A cells (Figure 1A and B). Next, an MTT assay showed that NF-κB overexpression increased HeLa and C_33_A cell viability compared with the control group at 24, 48, and 72 h after cell seeding (Figure 1C). Conversely, the knock down of NF-κB suppressed cell viability (Figure 1D). The colony formation assay also demonstrated that NF-κB overexpression increased HeLa and C_33_A colony numbers by approximate 25% and 40%, respectively, compared with the control vector (Figure 1E), but decreased NF-κB levels reduced the colony numbers (Figure 1F). These results indicate that NF-κB promotes cervical cancer cell viability and growth.

### NF-κB promotes human cervical cancer cell growth through miR-130a

**Figure 2:**
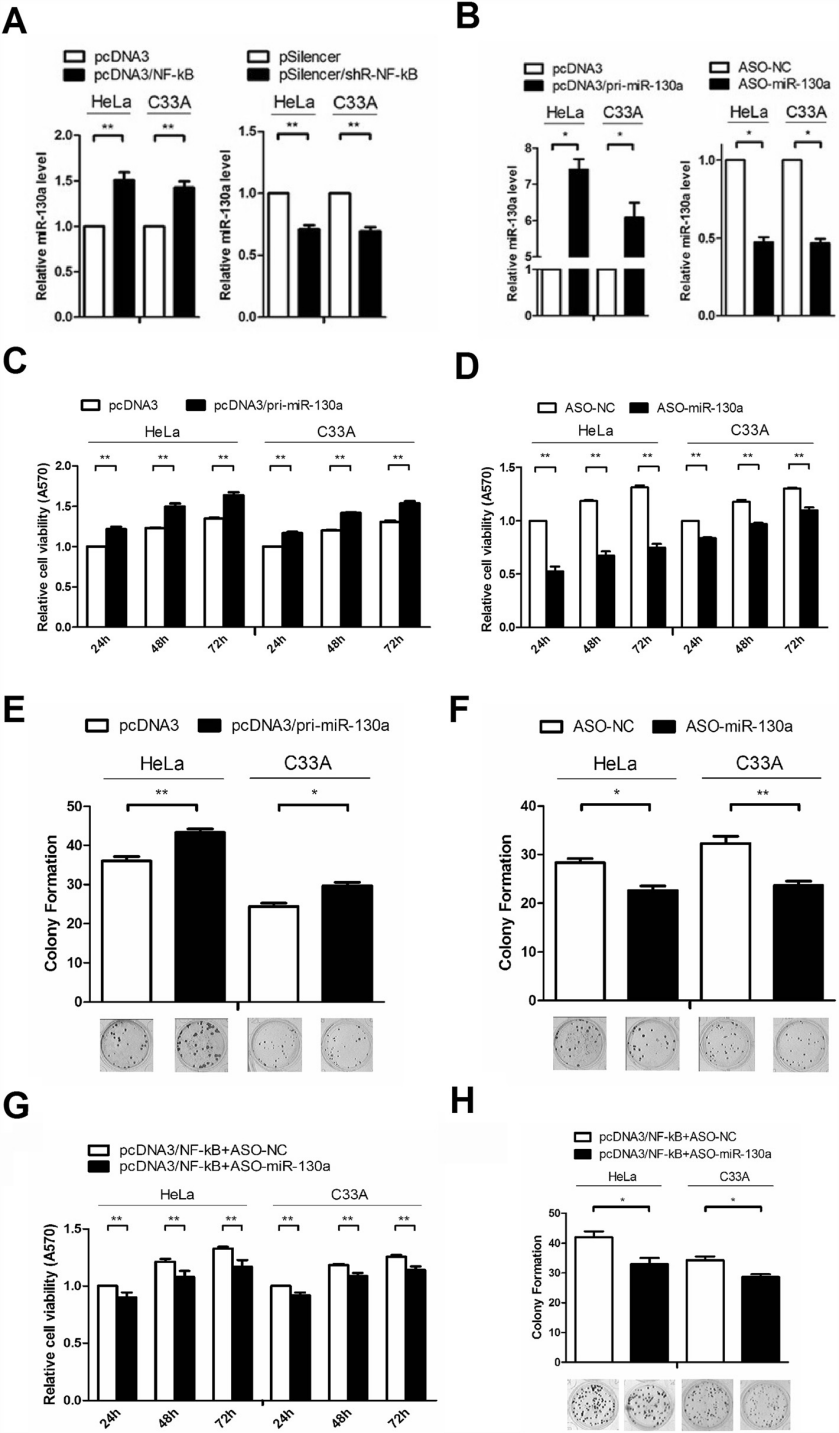
**miR-130a promotes the growth of human cervical cancer cells. (A)** The effects of NF-κB on miR-130a expression. Real-time RT-PCR was used to evaluate miR-130a levels in HeLa and C_33_A cells transfected with the pcDNA3/NF-κB or pSilencer/shR-NF-κB plasmids. Total RNA was extracted 24 h post-transfection and used for reverse transcription and real-time PCR. U6 snRNA was used to normalize gene expression. **(B)** Real-time RT-PCR was performed to detect the miR-130a levels in the two cell lines treated with pri-miR-130a or ASO-miR-130a. U6 snRNA was used to normalize gene expression. **(C and D)** MTT assays were performed to determine the effects of miR-130a on cell viability. HeLa and C_33_A cells were detached from 24-well plates after transfection with pcDNA3/pri-miR-130a or a control vector and ASO-miR-130a or control oligonucleotides, and the relative cell growth was determined at 24, 48 and 72 h after seeding in 96-well plates. **(E and F)** The effects of miR-130a on cell proliferation were evaluated by a colony formation assay. Cells were detached from 24-well plates after transfection and seeded in 12-well plates. On the 14^th^ day after seeding, the number of colonies was determined. **(G and H)** MTT and colony formation assays using by NF-κB in cells with miR-130a knockdown. Experiments were performed in triplicate. (*P < 0.05, **P < 0.01).

In our previous experimental work, gene chip results showed some miRNAs which may regulated by NF-κB, miR-130a was in them and relatively few existing reports about it, so we picked it as our object of study for a further explore. When HeLa and C_33_A cells were transfected with pcDNA3/NF-κB or pSilencer/shR-NF-κB, qRT-PCR indicated that NF-κB overexpression increased the miR-130a levels by approximately 50% and 40% compared to that of control vector, but a knock down of NF-κB decreased the miR-130a levels by 25% in both HeLa and C_33_A cells, respectively (Figure 2A). These results indicate that NF-κB may induce miR-130a expression.

Some reports have shown that miR-130a can mediate endothelial cancer cell growth [40,41], and our data also showed that NF-κB enhanced the growth of cervical cancer cells and induced miR-130a expression. Thus, we speculated that miR-130a may mediate NF-κB’s cell growth effects. First, we validated the efficiencies of pcDNA3/pri-miR-130a or miR-130a antisense oligomers (ASO-miR-130a). qRT-PCR revealed that cells transfected with pcDNA3/pri-miR-130a produced 7.5 and 6-fold increases in miR-130a levels. However, ASO-miR-130a reduced miR-130a levels by almost 50% in transfected HeLa and C_33_A cells (Figure 2B). Then, we performed MTT and colony formation assays in transfected HeLa and C_33_A cells and found that pcDNA3/pri-miR-130a increased cell viability and the colony formation rate by approximately 20% compared with the control group (Figure 2C and E). Moreover, ASO-miR-130a led to a suppression of cell viability and colony formation (Figure 2D and F) rate. These results indicate that miR-130a facilitates HeLa and C_33_A cell viability and growth, which is consistent with NF-κB’s effects on HeLa and C_33_A cells.As miR-130a and NF-κB plays the same role on the growth of cancer cells, which prompt us miR-130a maybe one of the regulatory mechanisms of NF-κB, in order to verify this, we performed an experiment using by NF-κB in cells with miR-130a knockdown. Results showed that as miR-130a knockdown, cell growth ability decreased compare with the group only overexpressed NF-κB both in the MTT and colony formation assays (Figure 2G and H).

### miR-130a directly targets and negatively regulates TNF-α expression

**Figure 3:**
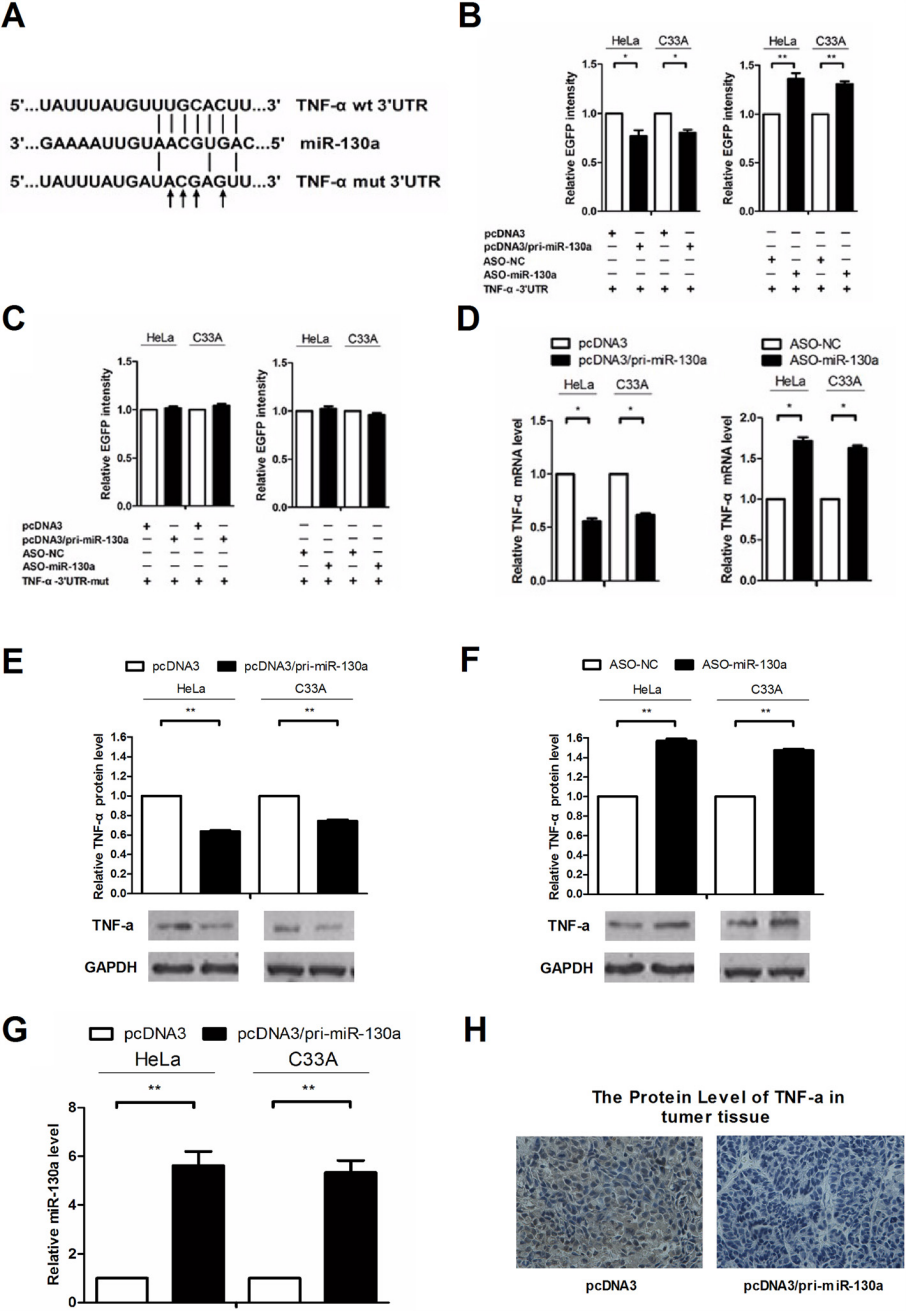
**miR-130a directly targets and negatively regulates TNF-α expression. (A)** Wild type (wt) and mutant complementary TNF-α mRNA 3’UTR sequences are shown compared with the miR-130a sequence. **(B and C)** EGFP reporter assays were performed to confirm the direct interaction of miR-130a and the TNF-α 3’UTR. HeLa and C_33_A cells were transfected with an EGFP reporter plasmid and the pcDNA3/pri-miR-130a or ASO-miR-130a plasmids, and the EGFP intensity was measured. **(D)** In HeLa and C_33_A cells, TNF-α expression levels were measured by real-time RT-PCR. The endogenous expression levels of β-actin mRNA were used to normalize mRNA expression. **(E and F)** Alterations in the TNF-α protein levels. miR-130a was overexpressed or its endogenous expression was blocked in both cell lines, and total protein was harvested for Western blot analysis. GAPDH protein expression levels were used to normalize the protein expression data. **(G)** HeLa and C_33_A cells were transfected with pcDNA3/pri-miR-130a or control vector and then implanted subcutaneously in the right armpit of nude mice, collected tumor after 14 days and made real-time RT-PCR assay to detect the expression levels of miR-130a in tumors. U6 snRNA was used to normalize gene expression. **(H)** Immunohistochemistry detected the levels of TNF-α protein in cervical cancer overexpress miR-130a compare with the control group. The histograms show the normalized mean ± SD mRNA levels and protein intensities from three independent experiments. (*P < 0.05, **P < 0.01).

miRNAs regulate a number of cellular functions through the downregulation of target gene expression by binding to 3’UTRs. To predict candidate target genes of miR-130a, we used three algorithm programs, TargetScan, PicTar, and miRanda. Using these programs, we selected TNF-α as a miR-130a target gene for further study because of its ability to suppress cancer cell growth and to activate NF-κB. The complementary sequence between the seed sequence of miR-130a and the TNF-α 3’UTR is shown in Figure 3A. To elucidate miR-130a’s direct regulation of TNF-α, an enhanced green fluorescent protein (EGFP) reporter assay was used to identify the target site in the TNF-α 3’UTR. We first constructed an EGFP reporter plasmid by inserting the miR-130a binding site in the TNF-α 3’UTR downstream of the EGFP stop codon (pcDNA3/EGFP-TNF-α-3’UTR) and a mutant seed sequence in a reporter plasmid (pcDNA3/EGFP-TNF-α-3’UTR-mut). Next, HeLa and C_33_A cells were cotransfected with the pcDNA3/EGFP-TNF-α-3’UTR reporter plasmid and pcDNA3/pri-miR-130a or ASO-miR-130a plasmids. We found that pcDNA3/pri-miR-130a expression led to a 25% decrease in EGFP fluorescence intensity compared with the control group, and the EGFP fluorescence intensity in cells transfected with ASO-miR-130a increased more than 25% (Figure 3B). However, there were no significant changes in the fluorescence intensities of the pcDNA3/EGFP-TNF-α-3’UTR-mut group regardless of altered miR-130a levels (Figure 3C). Furthermore, we examined endogenous TNF-α that had been downregulated by miR-130a. Overexpression of miR-130a led to an approximately 50% reduction in TNF-α mRNA levels, but conversely, blocking miR-130a expression increased TNF-α mRNA levels by approximately 50% (Figure 3D). Western blot analysis showed that pcDNA3/pri-miR-130a reduced TNF-α protein expression levels by 40% in HeLa cells and 30% in C_33_A cells (Figure 3E), and blocking endogenous miR-130a expression increased TNF-α protein levels by 50% in both cells types (Figure 3F). We also verified this result in vitro, cells were transfected with pcDNA3/pri-miR-130a and its control vector then injected subcutaneously in the flank and collected the tumor tissue. In the RNA extracted from tissue, miR-130a was increased compared to the control group (Figure 3G), and staining of TNF-α was decline (Figure 3H). Altogether, these results indicate that miR-130a can bind to the TNF-α 3'UTR and negatively regulate its mRNA and protein expression levels.

### TNF-α overexpression counteracts the effects of miR-130a

**Figure 4:**
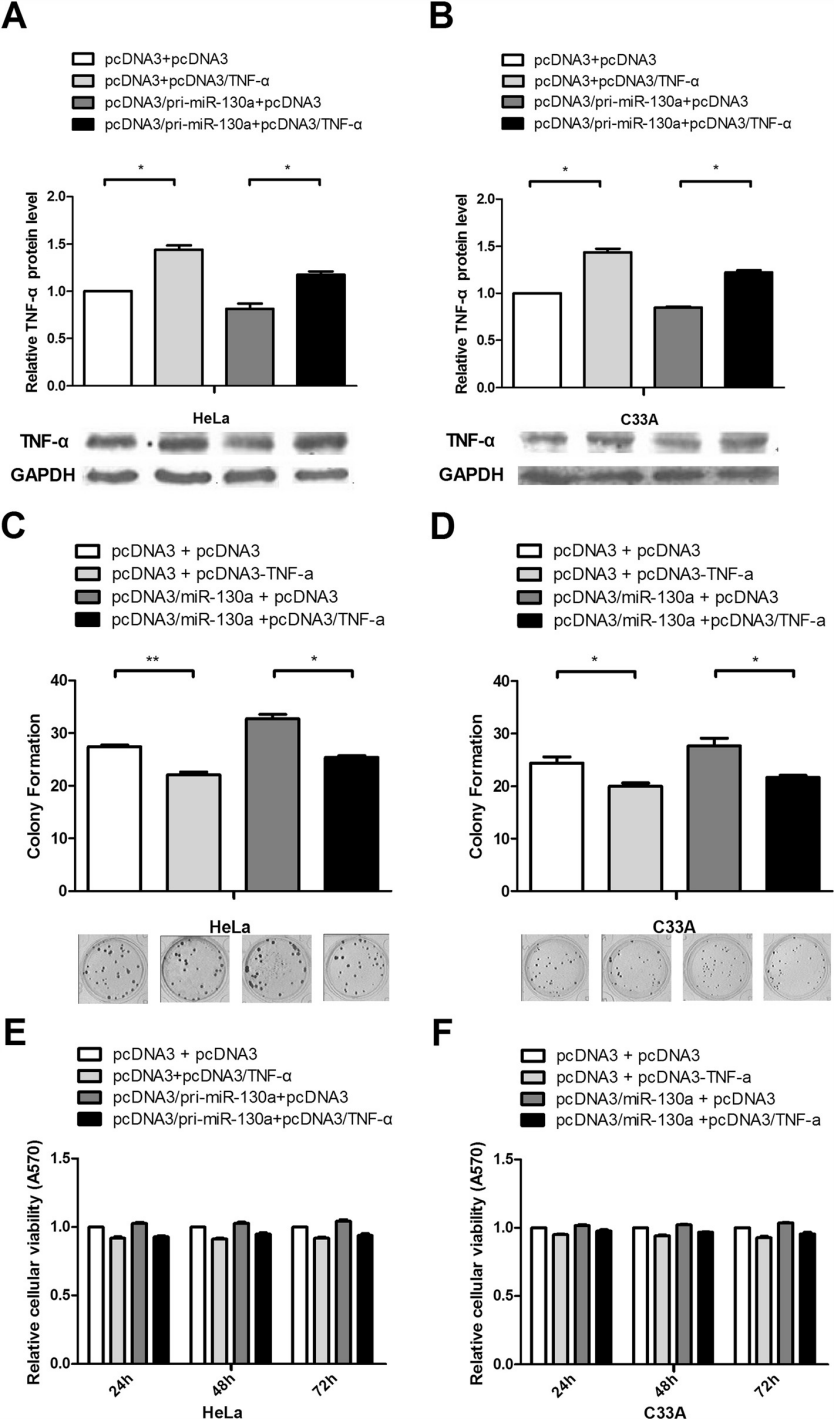
**TNF-α overexpression counteracts the effects of miR-130a. (A and B)** Western blot analysis was used to determine TNF-α protein levels and validate the TNF-α overexpression vector. HeLa and C_33_A cells were co-transfected with the pcDNA3/TNF-α plasmid, which did not contain the TNF-α 3’UTR, with or without the pcDNA3/pri-miR-130a plasmid. GAPDH protein expression was used to normalize the endogenous expression data. **(C and D)** Colony formation assays were performed to determine the effects of TNF-α on cell proliferation. We transfected the pcDNA3/TNF-α vector with or without cotransfection of pcDNA3/pri-miR-130a as in Figure 2G and H. **(E and F)** MTT assay used to assess cell viability. The data represent the mean ± SD of three independent experiments. (*P < 0.05, **P < 0.01).

To confirm that miR-130a promotes cervical carcinoma cell growth through at least a partial downregulation of TNF-α, we generated a TNF-α expression vector (pcDNA3/TNF-α) lacking the TNF-α 3’UTR to minimize miRNA interference. miR-130a was overexpressed with TNF-α in HeLa and C_33_A cells, and then growth activity was examined by MTT and colony formation assays. Western blot analysis showed that the miR-130a-induced reduction in TNF-α levels was rescued by the pcDNA3/TNF-α plasmid transfected into HeLa and C_33_A cells (Figure 4A and B). In the colony formation assay, ectopic TNF-α expression abrogated the cell growth enhancement caused by miR-130a compared with the control vector (Figure 4C and D), but in the MTT assay, no obvious changes were observed between the experimental and control groups (Figure 4E and F). Therefore, these results provide further evidence that TNF-α overexpression counteracts the repressive effects of miR-130a on cell growth.

### TNF-α stimulates NF-κB to upregulate miR-130a expression to form a TNF-α/NF-κB/miR-130a feedback loop

**Figure 5:**
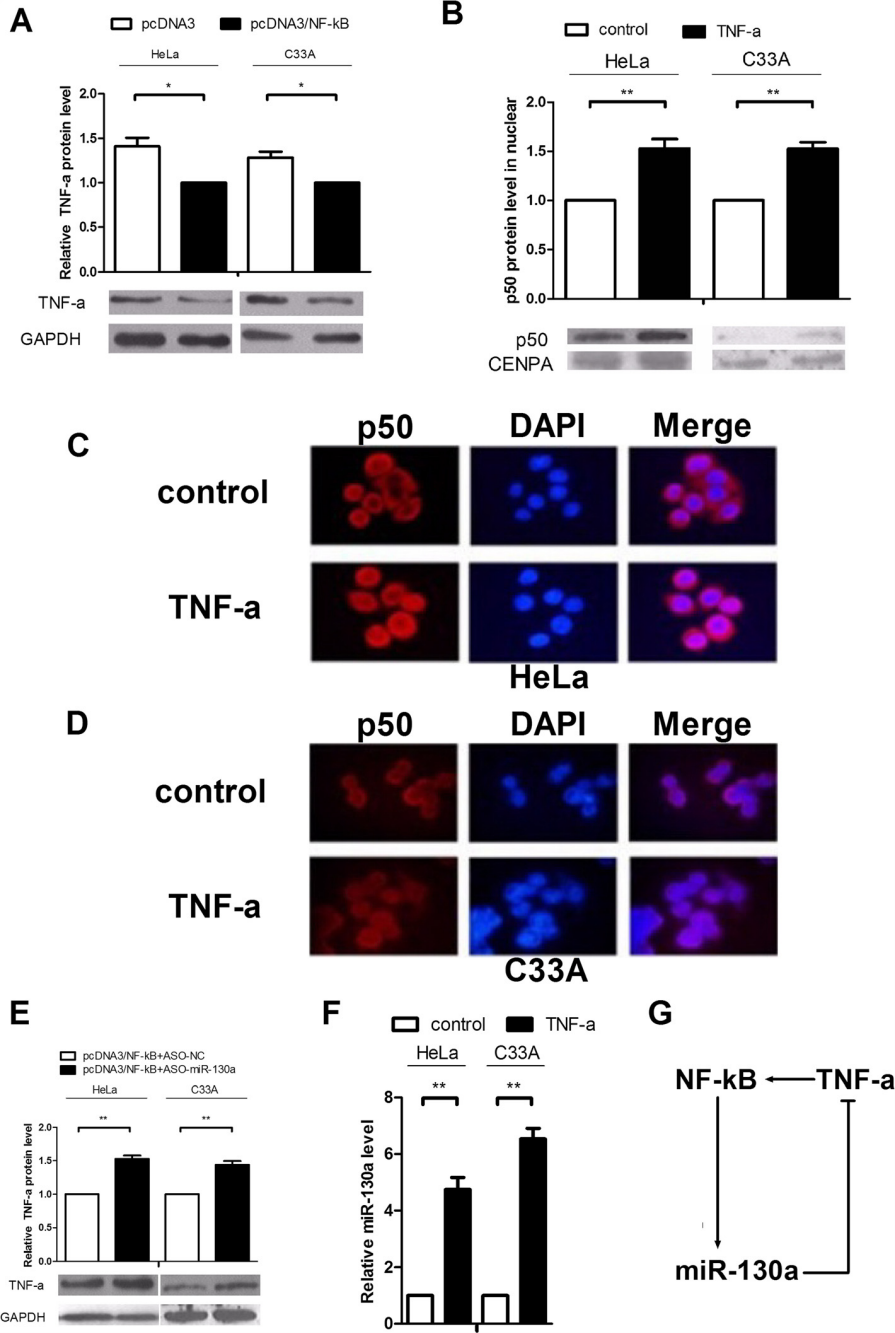
**NF-κB inhibits TNF-α expression, and TNF-α increases NF-κB activity. (A)** HeLa and C_33_A cells were transfected with pcDNA3/NF-κB, and total protein from cells was collected to determine the TNF-α levels. The endogenous GAPDH expression levels were used to normalize protein expression. **(B)** Cells were treated with TNF-α, and Western blot analysis was used to detect NF-κB activity. Cells were collected in PBS, centrifuged, resuspended in 0.4% NP-40, and centrifuged again to obtain nucleoproteins. Nuclear protein CENPA were used to normalize protein expression. **(C and D)** Cells were seeded on 14-well slides and stimulated with TNF-α (20 ng/mL) for 24 h. Next, cells were fixed with 4% paraformaldehyde, permeabilized with 0.5% Triton X-100, and blocked with 10% donkey serum. A primary antibody raised against p50 was diluted in 1% normal donkey serum and incubated at 4°C overnight. Cells were washed, and a FITC-labeled donkey anti-rabbit antibody was diluted in 1% normal donkey serum. Finally, DAPI was used to stain the nuclei. All the experiments were performed in triplicate. **(E)** Verify the role of miR-130a in the NF-kB induction of TNF-α. Cells were transfected with pcDNA3/NF-κB and ASO-miR-130a compare pcDNA3/NF-κB and ASO-NC, the cells were collected totle protein for Western Blot. GAPDH protein expression was used to normalize the endogenous expression data. **(F)** Cells were treated with TNF-α (20 ng/mL) for 24 h, and RNA was extracted for real-time RT-PCR. U6 snRNA was used to normalize gene expression. **(G)** The relationship between NF-κB, miR-130a and TNF-α. (*P < 0.05, **P < 0.01).

TNF-α is a strong activator of NF-κB [42,43], and our findings indicated that NF-κB could stimulate miR-130a expression, which in turn downregulated TNF-α expression (Figure 5A). Therefore, we speculated that TNF-α may regulate miR-130a expression through the NF-κB pathway. Twenty-four hours after TNF-α treatment, we extracted cytoplasmic and nuclear proteins from HeLa and C_33_A cells to detect NF-κB and found that the nuclear p50 content was significantly increased as assessed by Western blot (Figure 5B). Immunofluorescent staining also showed that TNF-α increased nuclear p50 (red) levels with compared with the control (Figure 5C and D). In Figure 2G and H, we seen NF-κB increased the growth capacity of crvical cncer cells through miR-130a, and we have demonstrated that miR-130a can direct targeting TNF-α, so we made a transfection of ASO-miR-130a and NF-κB, we found that the consume of miR-130a rescue the reduction of TNF-α inducted by NF-κB (Figure 5E), these results strongly support our hypothesis. Moreover, TNF-α treatment upregulated miR-130a levels by approximately 5-6.5-fold in HeLa and C_33_A cells (Figure 5F). Altogether, TNF-α initially stimulated NF-κB activation to induce miR-130a expression, which in turn targeted and down-regulated TNF-α expression and suggests a TNF-α/NF-κB negative feedback loop acting through miR-130a in cervical cancer cells (Figure 5G).

## Discussion

NF-κB is one of the most important intracellular nuclear transcription factors, and it plays a central role in the transcriptional regulation of many genes that are influenced by various stimuli. Some studies have shown that NF-κB promotes tumorigenesis through transcriptional regulation. NF-κB activation is involved in Ras-induced carcinogenic effects and telomerase reverse transcriptase activity [44-46], but it can also promote apoptosis [47-49]. Here, utilizing MTT and colony formation assays, we demonstrated that NF-κB played a growth-promoting role in the HeLa and C_33_A human cervical cancer lines.

miRNAs have been found to be involved in physiological and pathological processes. Moreover, NF-κB has been shown to regulate miRNA expression, and in turn, some miRNAs modulate NF-κB expression directly or indirectly [50,51]. We are interested in miRNA regulation of the NF-κB signal pathway. Hence, we found that NF-κB induced miR-130a expression to significantly increase, whereas the knockdown of NF-κB repressed miR-130a expression. Therefore, we conclude that NF-κB promotes miR-130a expression. Numerous studies have shown that miR-130a plays an important role in cell function and carcinogenesis by targeting various genes, such as FOG-2 [36], GAX [37] and Smad4 [38] in cardiomyocytes, vascular smooth muscle cells and granulocytic precursors cells, respectively. In cancer, miR-130a targets MET in non-small cell lung carcinoma [40] and can target ATG2B and DICER1 to kill Chronic Lymphocytic Leukemia cells [41]. To determine whether miR-130a’s effects on cervical cancer growth are downstream of NF-κB, we used gain-of- and loss-of-function assays to examine the role of miR-130a in cervical cancer cells. MTT and colony formation assays showed that miR-130a promoted HeLa and C_33_A cell viability and colony formation compared with the control, which is consistent with NF-κB’s ability to enhance the growth of human cervical cell lines.

miRNA possesses diverse roles that up- or down-regulate target gene expression [52-57]. To identify the miR-130a target genes responsible for its effects on cervical cancer cells, we used bioinformatics and functional knowledge associated with NF-κB and miR-130a and chose TNF-α as a candidate gene for further study. In the EGFP reporter assay, the expression of an EGFP reporter plasmid containing the TNF-α 3’UTR was repressed by miR-130a, and the mutated TNF-α 3’UTR abolished this effect. Furthermore, qRT-PCR and Western blot analysis showed that miR-130a decreased TNF-α mRNA and protein expression levels in cervical cancer cells compared with the control. Together, these data suggest that miR-130a downregulates TNF-α expression by binding to its 3’UTR. Moreover, ectopic TNF-α expression lacking the 3’UTR abrogated the effects of miR-130a on cervical cancer cell growth in the colony formation assay, but cell viability assessed by MTT assay was not obviously affected. These effects may result from miR-130a’s regulation of TNF-α requiring a certain length of time to be effective or involving other target genes; however, the detailed mechanism still needs to be investigated. Therefore, we conclude that miR-130a promotes cell growth through at least the partial involvement of TNF-α.

Tumor necrosis factor-alpha has been reported to be related to a variety of physiological processes, including cytotoxicity and the regulation of anti-viral and immune responses, and most of these processes are regulated through downstream regulatory factors [58,59]. Although TNF-α can destroy tumors, in different environments, it may possess other effects, even acting as an endogenous tumor promoter [27-29,60]. TNF-α is a strong NF-κB activator [61-63]. While high concentrations of TNF-α can induce the death of cervical cancer cells [64-66], we found that low TNF-α concentrations induced cervical cancer cell growth. However, how NF-κB provides feedback regulation of TNF-α expression is not well known. Here, we found that TNF-α stimulated the nuclear translocation of NF-κB and induced miR-130a expression in HeLa and C_33_A cells. A recent report showed that LPS could increase miR-130a expression, and NF-κB was involved in this process in human biliary epithelial cells [11]. Because LPS is a widely known TNF-α activator, these results support our conclusions. Thus, TNF-α stimulates NF-κB to induce miR-130a expression, and in turn, miR-130a downregulates TNF-α expression, which may form a feedback loop of TNF-α/NF-κB/miR-130a/TNF-α. This negative feedback loop may regulate TNF-α production to maintain relatively low concentrations. These low TNF-α concentrations may stimulate the nuclear translocation of NF-κB and enable cells to survive. Therefore, this NF-κB/miR-130a/TNF-α feedback loop may contribute to low TNF-α concentrations that avoid the induction of apoptosis in carcinogenesis.

## Conclusions

This study is the first to demonstrate that NF-κB and miR-130a can promote the growth of human cervical cancer cells and identifies TNF-α as a new target gene of miR-130a. NF-κB can increase miR-130a expression, and this enhancement of miR-130a expression inhibited TNF-α expression, which is a direct target of miR-130a. Low levels of TNF-α induced nuclear NF-κB translocation, which caused a gradual decline in miR-130a expression. Based on these results, we provide evidence of the existence of an NF-κB/miR-130a/TNF-α/NF-κB feedback loop in cervical cancer cells (Figure 5G). Our experimental results may aid in the understanding of the molecular mechanisms involving NF-κB and tumorigenesis and might provide a new potential biomarker of diagnostic and therapeutic value for cervical cancer patients.

## Abbreviations

ASO: Antisense oligonucleotide; EGFP: Enhanced green fluorescence protein; GAPDH: Glyceraldehyde-3-phosphate dehydrogenase; UTR: Untranslated region; qRT-PCR: Quantitative Reverse Transcription-PCR.

## Competing interests

The authors declare no competing interests.

## Authors’ contributions

JZ: participated in the experimental work and preparation of manuscript. HW, PL, YZ: participated in the experimental work. Ml: analyzed the data and participated in article preparation. HT: designed, directed the experiment. Analyzed data and prepared the manuscript. All authors read and approved the final manuscript.
